# Clinical Challenges in a 49-Year-Old Patient with Severe Tick-Borne Myeloradiculitis Despite Complete Active Vaccination

**DOI:** 10.3390/vaccines8010093

**Published:** 2020-02-20

**Authors:** Julia Feige, Tobias Moser, Larissa Hauer, Slaven Pikija, Johann Sellner

**Affiliations:** 1 Department of Neurology, Christian Doppler Medical Center, Paracelsus Medical University, 5020 Salzburg, Austria; j.feige@salk.at (J.F.); t.moser@salk.at (T.M.); s.pikija@salk.at (S.P.); 2 Department of Psychiatry, Psychotherapy and Psychosomatic Medicine, Christian Doppler Medical Center, Paracelsus Medical University, 5020 Salzburg, Austria; l.sellner@salk.at; 3 Department of Neurology, Klinikum rechts der Isar, Technische Universität München, 81675 München, Germany; 4 Department of Neurology, Landesklinikum Mistelbach-Gänserndorf, 2130 Mistelbach, Austria

**Keywords:** vaccination failure, tick borne encephalitis, myeloradiculitis, outcome

## Abstract

Vaccination is an effective means to prevent infectious diseases including tick-borne encephalitis (TBE), an emerging Flavivirus infection. There is, however, only limited knowledge about risk of vaccination failure, the disease course and the challenges for work-up and care. Of note, there is evidence that patients with breakthrough disease experience a more severe disease course. We report the case of a previously healthy 49-year-old woman who developed severe myeloradiculitis caused by the TBE virus despite receiving a complete cycle of primary immunization and booster vaccinations within the recommended timeframe. The disease course was characterized by progressive tetraparesis, pain and bladder dysfunction and necessitated intensive care unit admission (ICU) and the escalation of pain management. This case raises awareness for the recognition of breakthrough disease in younger patients and reinforces the need to develop measures to identify patients with insufficient protection.

## 1. Introduction

Tick-borne encephalitis (TBE) is an emerging Flavivirus infection with 10,000-12,000 cases reported in Europe each year [1]. The highest incidences of clinical cases are reported from the Baltic States, Slovenia and the Russian Federation. Most infections result from tick bites which were sustained during outdoor activities in forested areas. The median incubation period is 8 days (range, 4–28 days) after the tick bite [2]. The typical disease course is biphasic. The first stage is characterized by a few days of non-specific symptoms including fever, fatigue and body pain. After a symptom-free week, a subgroup of infected persons develops acute central nervous symptoms (CNS) along with fever [3]. These include aseptic meningitis (50%), meningoencephalitis (40%) and myelitis (10%) [2]. Poliomyelitis-like illnesses or radicular involvement are characteristic of these conditions, together with significant persistent neurological sequelae [4]. Some patients develop respiratory complications and require treatment at the intensive care unit (ICU) [5]. The mortality rate of European cases continues to be in the range of 1–2%.

Vaccination is an effective measure to prevent morbidity and mortality associated with infectious diseases. This is also the case for TBE where no specific therapy is approved and treatment is supportive only [6]. TBE is also endemic in all Austrian federal states and a vigorous vaccination campaign launched in 1981 can be regarded as a continuing success story [7]. In this regard, up to 85% of the population received a primary vaccination course so far and the incidence dropped from 5.7 to 0.9 cases per 100.000 [8]. There is, however, increasing evidence about the occurrence of immunization failure with the TBE virus (TBEV) vaccine. A comprehensive study on TBE in Europe (5205 cases) reported that the condition developed in 87 patients (1.7%) despite those individuals receiving at least two primary vaccination courses [9]. The European vaccines are licensed for adults and children >1 years [6]. They require a primary series of three doses. Those who will continue to be at risk should have ≥1 booster doses. A major risk factor for developing a breakthrough infection is both having received two instead of three primary vaccination courses and older age [10,11].

We report the case of a previously healthy 49-year-old woman who developed severe myeloradiculitis caused by the TBE virus despite receiving a complete cycle of a primary vaccination and booster vaccinations within the recommended timeframe.

## 2. Case Report

### Case Study

A 49-year-old afebrile woman sought treatment at our department due to intractable lower back pain and a mild paresis of the right leg. The symptoms had increased over the course of four days; there was no prodromal phase. Her medical history included an early localized borrelia infection nine years earlier, which was treated with a full course of third generation cephalosporins. She did not take regular medication but had received analgesics (metamizole, diclofenac) from her general practitioner. Living in an endemic region and being at risk of disease exposure as a part-time farmer, she had been immunized for TBEV with a 3-dose standard scheme in childhood and received booster vaccinations according to the national recommendations for individuals at higher risk since then (every three years).

**Figure 1:**
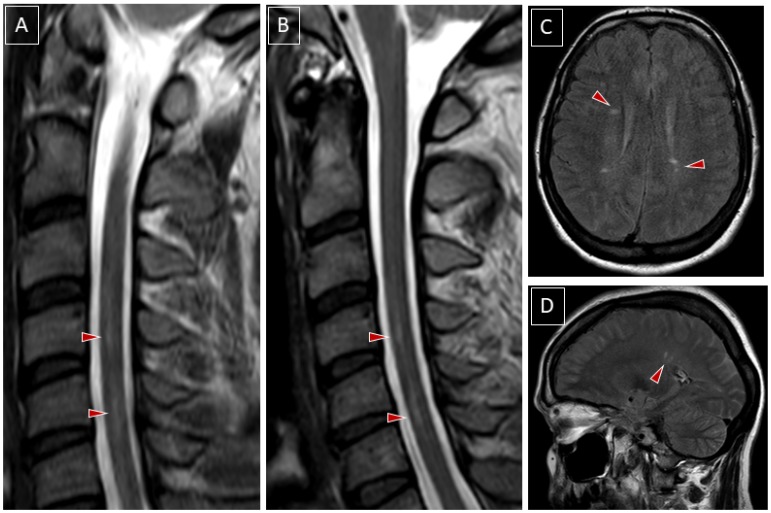
Radiological features of longitudinally extensive myelitis related to the tick-borne encephalitis virus (TBEV) which caused a Polio-like illness. Panel (**A**). Spinal cord MRI T2 sagittal images in the acute phase. The presence of a tractopathy involving the central and anterior section of the cervical spinal cord which expands over more than three vertebral bodies is depicted by red arrows. Panel (**B**). A follow-up scan one month later demonstrates a slight reduction of the hyperintense signal, shown by the red arrows. Panels (**C**,**D**). Fluid-attenuated Inversion recovery (FLAIR) MRI (axial and sagittal planes) revealed several oval-shaped hyperintensities perpendicular to the ventricle system, consistent with white-matter disease, shown by the red arrows.

A T2-weighted MRI of the spinal cord revealed hyperintense signals in the central and anterior plane of the cervical to the midthoracic cord (Figure 1). Patchy contrast enhancement was detected between C3 to Th2 levels. She was administered antibiotic treatment with iv ceftriaxone 2g daily as well as antiviral therapy with acyclovir 10mg per kg bodyweight every 8 hours after CSF diagnostics revealed a mild lymphomononuclear pleocytosis (65 cells/µL, <4), elevated protein levels (84 mg/dl, 3–50) and increased lactate (3.1 mmol/l, 1.1–2.4). There was an intrathecal IgG synthesis (28%) and a presence of CSF-specific oligoclonal bands (OCB). A brain MRI revealed a couple of non-enhancing white matter lesions, partly in association with the corpus callosum and in the periventricular vicinity. There was an intrathecal IgG production of the Varicella-zoster virus (2.1, <1.5) but the PCR of the CSF specimen was negative. The PCR testing for other Herpes viruses in CSF and the work-up for borrelia were negative (negative antibody index and PCR, and CXCL13 values within normal limits). We did not perform PCR for the TBEV in CSF.

Additional serological tests were directed towards exclusion of neuromyelitis spectrum disorders (aquaporin-4 (AQP4) and myelin oligodendrocyte glycoprotein (MOG) antibodies), other neurotropic viruses (West-Nile Virus (WNV)) and CNS manifestations of systemic autoimmune disorders which included serological tests of autoimmune and paraneoplastic antibodies, dermatological and rheumatological consultation and additional imaging.

Three days later, she developed voiding dysfunction, paresis of the left arm and paraplegia of the lower extremities. The rapid progression of motor symptoms necessitated a temporary transfer to the intensive care unit (ICU) but eventually ventilation was not required. The treatment of pain was further escalated with gabapentin, piritramide and fentanyl.

There was no evidence for the aforementioned conditions as a cause of longitudinally extensive transverse myelitis (LETM) and repeat CSF examination revealed a further increase of the cell count (75 cell/µL, predominantly lymphomononuclear cells), elevation of CSF protein (174 mg/dL) and IgG synthesis (48%). We thus extended the work-up towards TBE with the possibility of vaccination failure in mind. Serological tests were reactive for TBEV (positive IgM in both serum and CSF, IgG initially 13,679 VIE in serum and after one week, 24,600 VIE units in serum and >1000 VIE units in CSF) and corroborated the diagnosis of myeloradiculitis due to the TBEV despite active immunization and regular booster vaccinations. The neurological deficits improved at the time of relocation to a specialized rehabilitation facility and she was able to manage the transfer by herself.

On clinical examination two years later, a mild lower extremity paraparesis persisted whereas the function of the upper extremity, urinary dysfunction and pain had fully recovered. A brain MRI performed twice over a period of 2 years showed neither new or expanding lesions nor contrast enhancement.

## 3. Discussion

Vaccination failure for the TBEV has been characterized in-depth only recently. Thus, there is no consensus for the criteria for vaccine failure but the definition of “A clinical case of TBE with debut of symptoms after at least two doses of vaccine. The last dose of vaccine received within (i) >4 weeks and <12 weeks if two vaccine doses, (ii) <36 months if three vaccine doses and (iii) <60 months if four or more vaccine doses” could be used as guidance [11]. Our patient fulfilled the latter and was classified as a confirmed case according to the European Academy of Neurology consensus paper by the detection of TBE-specific IgG and IgM in serum, increase of IgG in serum and IgM in CSF [6]. Of note, the presumed protection suggested by the history of complete active vaccination should not detain from diagnostic work-up. A similar observation is mentioned in a report of a fatal TBE breakthrough case with myeloradiculitis from Switzerland [12]. The neuropathological analysis revealed a predominantly T-cell-driven parenchymal infiltration, which implicates that the vaccine-induced T-cell and antibody response failed and subsequently allowed the virus to invade the brain parenchyma and the spinal cord. Interestingly, the histopathological picture indicates direct motor neuron damage by the virus and, less dominantly, indirect damage by an inflammatory response.

Using the aforementioned criteria, a recent Swedish study evaluated a total of 1004 TBE cases and defined 53 of them (5%) as vaccine failures [11]. These data correspond to the previous Austrian estimates for the field efficacy of vaccinations against the European TBEV subtype of 96–99% [13]. In 2018, Lenhard and coworkers summarized the 11 cases available in the literature on European TBE despite vaccination history and found 10 cases of irregular vaccination schedule and one case of breakthrough disease [14]. Interestingly, these patients showed a significantly stronger cellular immune response as measured by CSF pleocytosis (irregular vaccination schedule, 205 cells/μL versus non-vaccinated control, 114 cells/μL). TBE-specific IgG was detected in our patient as evidence for previous vaccination. TBE-specific IgM can be negative early in the course, e.g., at the onset of symptoms. Therefore, the analysis of a single serum sample is insufficient for the exclusion of acute TBEV infection [15].

Our finding of extensive intrathecal IgG-synthesis and detection of OCB in combination brain lesions within the white matter and in vicinity to the corpus callosum led to additional diagnostic considerations. Yet, LETM is a rarity in MS and a tractopathy is not reported in MS. Thus, autoimmune conditions as a cause of LETM and viruses leading to anterior horn tractopathy including WNV and Herpes viruses needed to be excluded [16,17]. A similar observation to our CSF findings was made by the Swiss case where massive intrathecal IgM (92%) and IgG (73%) production was present [12]. In that patient, the combination of a history of a completed course of vaccination, the early serology results consistent with the vaccination and the rare incidence of vaccine failures markedly delayed the diagnosis.

Our patient had a monophasic course. This is a typical feature of patients with vaccination failure, as reported in 77% of patients in the Swedish cohort, which is more frequent in vaccinated patients [11]. The authors explain this observation by the presence of still circulating antibodies leading to an attenuated, and therefore subclinical, viremia. Several case reports had suggested a more severe disease course in patients with vaccination failure, which could be related to advanced age [10,12,15,18,19,20,21,22,23]. Indeed, vaccination failure is more frequent in older patients, as confirmed in the Swedish cohort, which had a median age of 62 years (range 6–83) [11]. Severe and moderate TBE disease affected 81% of the cases with vaccine failure and 6% died. Compared to unvaccinated controls, patients with vaccination breakthrough had a 2.65 increase in the risk of suffering from severe TBE. In detail, 81% of the patients were aged more than 50 years. Of interest, 26% had diseases with altered function of the immune system including malignancies and rheumatic diseases. Moreover, 15% of the patients were on immunosuppressive treatment. Vaccine failures following the third or fourth vaccine dose accounted for 36 (68%) of the patients. All of these issues, as well as higher age, did not pertain to our patient.

## 4. Conclusions

This case illustrates a typical presentation of anterior horn involvement and radiculitis related to TBE, as well as the hazards for the diagnostic work-up and interdisciplinary management in a relatively young patient despite the appropriate vaccination. Moreover, our report reinforces the need to better understand vaccination failure in TBE and develop measures to identify patients which have insufficient protection.
